# Optically Transparent Anionic Nanofibrillar Cellulose Is Cytocompatible with Human Adipose Tissue-Derived Stem Cells and Allows Simple Imaging in 3D

**DOI:** 10.1155/2019/3106929

**Published:** 2019-10-07

**Authors:** Jonathan J. Sheard, Mesude Bicer, Yiming Meng, Alessia Frigo, Rocío Martínez Aguilar, Thomas M. Vallance, Donata Iandolo, Darius Widera

**Affiliations:** ^1^Stem Cell Biology and Regenerative Medicine Group, School of Pharmacy, University of Reading, Whiteknights Campus, Reading RG6 6AP, UK; ^2^Sheard BioTech Ltd, 1st Floor Sheraton House, Lower Road, Chorleywood WD3 5LH, UK; ^3^Unidad de Inmunología, IBIMER, Universidad de Granada, Granada 18071, Spain; ^4^Department of Chemical Engineering and Biotechnology, University of Cambridge, Philippa Fawcett Drive, Cambridge CB3 0AS, UK

## Abstract

The anti-inflammatory and immunomodulatory properties of human mesenchymal stromal cells (MSCs) are a focus within regenerative medicine. However, 2D cultivation of MSCs for extended periods results in abnormal cell polarity, chromosomal changes, reduction in viability, and altered differentiation potential. As an alternative, various 3D hydrogels have been developed which mimic the endogenous niche of MSCs. Nevertheless, imaging cells embedded within 3D hydrogels often suffers from low signal-to-noise ratios which can be at least partly attributed to the high light absorbance and light scattering of the hydrogels in the visible light spectrum. In this study, human adipose tissue-derived MSCs (ADSCs) are cultivated within an anionic nanofibrillar cellulose (aNFC) hydrogel. It is demonstrated that aNFC forms nanofibres arranged as a porous network with low light absorbance in the visible spectrum. Moreover, it is shown that aNFC is cytocompatible, allowing for MSC proliferation, maintaining cell viability and multilineage differentiation potential. Finally, aNFC is compatible with scanning electron microscopy (SEM) and light microscopy including the application of conventional dyes, fluorescent probes, indirect immunocytochemistry, and calcium imaging. Overall, the results indicate that aNFC represents a promising 3D material for the expansion of MSCs whilst allowing detailed examination of cell morphology and cellular behaviour.

## 1. Introduction

The ability of MSCs to undergo multilineage differentiation, their regenerative capacity, as well as their anti-inflammatory and immunomodulatory properties, have led to an increase in their clinical application with over 913 trials registered on ClinicalTrials.gov as of January 2019.

Notably, the regenerative potential of MSCs observed in multiple preclinical and clinical studies is now widely believed to be a consequence of bystander effects that are mediated by extracellular vesicles rather than a result of differentiation and engraftment [1–4].

Despite their reported clinical performance, the wide application of MSCs is often hampered by the invasive isolation procedure if the cells are harvested from the human bone marrow. Thus, alternative sources of MSCs have been the focus of translational research including the adipose tissue where stem cells can be easily isolated within minimally invasive surgery [5]. In this context, it is widely known [6, 7] that ADSCs are readily harvested and isolated from adipose tissue with very low donor-site morbidity, whilst expressing typical mesenchymal cluster of differentiation (CD) markers. Additionally, ADSCs have been reported for their beneficial effects within multiple clinical applications including but not limited to chronic wounds [8] and osteoarthritis (reviewed by Damia et al. [9]), as well as secondary-progressive multiple sclerosis [10].

Nevertheless, culture expansion *in vitro* is still a necessary but costly step to obtaining sufficient quantities of cells for the intended therapeutic application. Notably, *in vitro* culture expansion of MSCs can lead to the accumulation of chromosomal aberrations [11], which may be due to the extraction of the cells from their endogenous niche [12]. Additionally, prolonged 2D cultivation has been reported to lead to a loss of multipotency and premature cellular senescence in MSCs [13].

To overcome these limitations of 2D cell culture, various 3D cultivation methods have been developed. Commonly used 3D cell carriers include, but are not limited to, alginate-based hydrogels [14, 15], bacteria-derived cellulose [16, 17], collagen-based matrices [18, 19], fibrin scaffold (Smart Matrix®), fibrin-poly(ester-urethane) scaffolds [20], and animal-derived basement membrane extracts (BMEs), such as mouse chondrosarcoma-derived Matrigel™ [21]. However, despite obvious advantages over 2D culture systems, 3D culture methods also have drawbacks. Notably, alginate hydrogels require cross-linking for gelation, where gel uniformity, mechanical properties, gel strength, and even the order of the network structure need to be very carefully monitored since these can be affected by the rate and temperature of gelation and the choice of cross-linking ions, as well as the chemical structure of the alginate itself [22–24]. Whilst these parameters can be advantageous for some applications, these can add extra levels of complexity and reduce the reproducibility of 3D cultivation.

Another common drawback in many 3D culture systems is the difficult retrieval of functional cells for downstream application. For example, the retrieval of cells from fibrin- and collagen-based matrices can only be achieved by using enzymes which may also affect mammalian cells, although some reports deny this negative effect [25]. Matrigel™, an extracellular matrix product derived from mouse chondrosarcoma tumors is known to be affected by batch-to-batch variability, cross species immunogenicity, and consequently, the lack of translational potential [26, 27].

Once an appropriate hydrogel has been chosen, another challenge can be successful imaging and image quantification of cells within the matrix. This is at least partly affected by the so-called spatial gel inhomogeneity [28] which is known to reduce the optical clarity of several hydrogels including polystyrene [29] and alginate hydrogels [30]. For many hydrogels, this physicochemical property results in relatively high light absorbance over the whole visible light spectrum and interferes with multiple microscopy-based methods including immunocytochemistry, live cell imaging, and calcium imaging.

Within this proof-of-concept study, we used multiple state-of-the-art methods to test the structural and visual properties as well as the biocompatibility of a novel anionic form of a plant-derived nanofibrillar cellulose-based hydrogel (aNFC) with human ADSCs.

## 2. Materials and Methods

The handling and preparation of aNFC with and without cells was performed analogous to the preparation of GrowDex® (NFC) as detailed in Azoidis et al. [31].

### 2.1. Nanofibrillar Cellulose and Calcofluor White Staining

The aNFC hydrogel (GrowDex®-T) and the cellulase enzyme (GrowDase™) were kindly provided by UPM Biomedicals (Helsinki, Finland). 0.2% *w*/*v* aNFC was prepared by diluting 1.0% stock solution in PBS (Sigma-Aldrich, Irvine, United Kingdom), and the hydrogel (100 *μ*l) was transferred into wells of a low-adhesion 96-well plate using low-retention pipette tips (Sarstedt Ltd., Leicester, United Kingdom) followed by the incubation with Calcofluor white (25 *μ*l, 0.01% *w*/*v*, Sigma-Aldrich) for 5 min at room temperature (RT). Calcofluor-stained aNFC was visualised using a DAPI filter on an inverted fluorescence microscope (VertA1, Carl Zeiss Ltd., Cambridge, United Kingdom).

### 2.2. Scanning Electron Microscopy

aNFC at 0.5%, with cells (1 × 10^6^ cells/ml) or without cells, was fixed with 2% formaldehyde and 2% glutaraldehyde in 0.05 M cacodylate buffer for at least 24 h before being rinsed. Hydrogels were washed twice with deionised water (DIW) to remove the fixative and placed on 10 mm Ø melinex coverslips (mesh-side down); excess water was removed. The samples were then plunge-frozen in liquid-nitrogen-cooled ethane. Samples were freeze-dried overnight in an Emitech K775X liquid-nitrogen-cooled freeze dryer (Quorum Technologies). Melinex coverslips were mounted on aluminium SEM stubs using silver DAG (TAAB); small amounts of silver-DAG around the bottom rim of the mesh/sample were used to secure the sample on the coverslip and ensure conductivity. Then, samples were coated with 35 nm gold and 15 nm iridium using an EMITECH K575X Sputter Coater (Quorum Technologies). Samples were viewed in a FEI Verios 460 scanning electron microscope at an accelerating voltage of 2 keV and a probe current of 50 pA. Images were acquired in a secondary electron mode using either an Everhart-Thornley Detector (ETD) or a Through-Lens Detector (TLD) in an immersion mode.

### 2.3. Image Analysis

SEM images of the 0.5% aNFC sample were used to measure the NFC fibre diameter and pore sizes using Fiji [32]. Over 280 individual fibres and pores were measured.

### 2.4. Light Absorbance Assay

GrowDex® (UPM Biochemicals, Finland) and aNFC were diluted to 0.2% using PBS and aliquoted into a 96-well plate. Total light absorbance in the ultraviolet, visible light, and infrared spectrum was measured between 240 and 800 nm using the SpectraMax iD3 plate reader (Molecular Devices, Wokingham, United Kingdom). Mean readings were corrected by subtracting PBS control values.

### 2.5. Human ADSCs

Fully characterised human adipose-derived MSCs from 5 nondiabetic adult donor lipoaspirates were obtained from Lonza (Slough, United Kingdom). All ADSCs have been characterised immunocytochemically as detailed by the manufacturer and as recommended by The International Society for Cellular Therapy [33].

### 2.6. Cultivation of ADSCs as 2D Monolayer

ADSCs were cultivated in standard cultivation medium prepared as follows:DMEM high glucose, 2 mM L-glutamine, 100 U penicillin with 100 *μ*g/ml streptomycin (all from Sigma-Aldrich), and 20% *v*/*v* heat-inactivated FBS (Sigma-Aldrich, lot: 8204188981) at 37°C and 10% CO_2_. Medium was changed every 2-3 days. All cells were used between passages 4 and 18.

### 2.7. Cultivation of ADSCs in aNFC 3D Matrix

As similarly detailed in Azoidis et al. [31], after detachment with 0.05% trypsin/EDTA (Sigma-Aldrich), ADSC suspensions were mixed with a 1.0% aNFC hydrogel to achieve a desired hydrogel concentration of 0.1%, 0.2%, 0.4%, or 0.5% (*w*/*v*) aNFC with cell densities of 1, 2.5, 5 × 10^4^ or 1 × 10^5^ cells/ml, respectively. After 30 min of incubation at 37°C, standard culture medium was added to the top of the hydrogel. Bright-field microscopy images were acquired using an inverted microscope (EVOS XL Cell Imaging System, Thermo Fisher Scientific).

For XTT viability assays, ADSCs were cultured in a 0.2% or 0.4% aNFC hydrogel in standard cultivation medium for up to 7 days at 37°C and 10% CO_2_. A 50% medium change was performed every 2-3 days.

Following 48 h incubation, retrieval of cells from aNFC was performed by enzymatic hydrolysis of the matrix with cellulase (600 *μ*g/mg) (GrowDase®, UPM Biomedicals) for 6 h at 37°C. After 6 h incubation, the solution was mixed multiple times to facilitate cell liberation and then centrifuged at 300*g* for 10 min. The cell pellet was resuspended in (50 *μ*l) media, and the cell count was performed using a haemocytometer and trypan blue (Sigma-Aldrich).

### 2.8. Cell Viability Assay (XTT)

Cell viability assays were performed for cells in both 2D and 3D cultures using the Cell Proliferation Kit II (Sigma-Aldrich) according to the manufacturer's instructions. Absorbance of the XTT metabolite was measured at an excitation wavelength of 490 nm and a reference wavelength of 650 nm on a SpectraMax iD3 plate reader (Molecular Devices, Wokingham, United Kingdom). Measurements were taken after 2 hours of incubation. Values were corrected for the reference values (650 nm), as well as the appropriate aNFC controls.

### 2.9. PKH67 Staining and Fluorescence Imaging

Living cells within the aNFC matrix were visualised using the PKH67 Green Fluorescent Cell Linker Kit for General Cell Membrane Labelling (Sigma-Aldrich). Briefly, MSCs (5 × 10^5^) were labelled according to the manufacturer's instructions and embedded within a 0.1% aNFC hydrogel. For the detection of the labelled cells, the CQ1 (Yokogawa Electric Corp., Japan) high-content confocal imaging system was used.

### 2.10. Live and Dead Staining and Confocal Laser Scanning Microscopy

ADSCs were cultured in 0.2% aNFC (1 × 10^6^ cells/ml) for 24 h within *x*-well cover glass cell culture chambers (Sarstedt Ltd.) and stained using LIVE/DEAD Viability/Cytotoxicity Kit according to the manufacturer's instructions (Thermo Fisher Scientific), fixed in 4% PFA for 20 min and counterstained with DAPI (Sigma-Aldrich). Confocal images were acquired using the Nikon A1R inverted confocal microscope with the Nikon Plan Apo VC 20x DIC N2 optic lens, running NIS-Elements AR. DAPI was visualised at an excitation/emission of 405/450 nm, calcein at 494/517 nm, and ethidium homodimer-1 at 528/617 nm with the Chroma 405/488/561/647 quad mirror. A *z*-stack depth of 200 *μ*m (*z*-plane) was created for an area of 554 × 550 *μ*m, and a 3D reconstruction was generated for all the channels using the NIS-Elements AR software (v4.0).

### 2.11. Adipogenic and Osteogenic Differentiation of ADSCs in 3D aNFC Hydrogels

For adipogenic and osteogenic differentiation, ADSCs (5 × 10^5^ cells/ml) in normal cultivation medium were embedded in 0.2% aNFC as described above and placed into TC cell culture inserts (24-well format, Ø3.0 *μ*M, 83.3932.300, Sarstedt Ltd.). Medium was changed after 24 h to StemPro Adipocyte or Osteocyte Basal Medium supplemented with StemPro Adipogenesis or Osteogenesis Supplement (Life Technologies) according to the supplier's instructions. For the controls, medium was changed to fresh standard cultivation medium. Control and differentiation media were replaced every 2-3 days and maintained at 37°C for 21 days. Following the differentiation period, media were removed from the wells and TC inserts. Cells were washed with PBS and subsequently fixed with 4% PFA for 30 min. For Oil Red O staining, 3 parts stock solution (300 mg Oil Red O in 100 ml 99% isopropanol, Sigma-Aldrich) was mixed with 2 parts deionised water then filtered. Following fixation, cells were washed with sterile double-distilled H_2_O. Staining solution was added to the cells for 5 min. For Alizarin Red staining, Alizarin Red staining solution (2 g Alizarin Red S in 100 ml distilled water, pH 4.1 with 0.1% NH_4_OH, filtered, Sigma-Aldrich) was added to cells and incubated for 45 min at RT in the dark. The staining solutions were removed and unbound dye was washed off by 5 washing steps with ddH_2_O. Images were taken using the EVOS imaging system.

### 2.12. Fluorescent Dye Staining

Phalloidin-Atto 555 (Sigma-Aldrich) was used to label the actin filaments of fixed ADSCs (1 × 10^5^ cells/ml) cultured in 0.2% aNFC within *x*-well cover glass cell culture chambers for 72 h. Following cell culture and PFA fixation as detailed above, ADSCs were gently washed with PBS for 5 min. The PBS was replaced with PBS/0.1% Triton X-100 (100 *μ*l) per well for 30 min at RT. The PBS/0.1% Triton X-100 was removed carefully, and the scaffold with cells was washed once with PBS for 5 min. Staining solution (100 *μ*l) was added to the cells and incubated overnight at 4°C. Staining solutions were prepared as follows: DAPI with phalloidin; DAPI working solution (0.5 *μ*g/ml) from a stock (1 mg/ml) by diluting it 1 : 2000 in sterile PBS; phalloidin working solution (0.2 nM) from a stock (10 nM) by diluting the stock with the DAPI working solution; Calcofluor with phalloidin; and phalloidin working solution (0.2 nM) was prepared from a stock (10 nM) by diluting it 1 : 50 with PBS. Calcofluor dye was diluted 1 : 33 with the phalloidin working solution. Following overnight incubation, the staining solutions were gently removed, and the cells were washed once with PBS. Following the final wash, PBS was replaced with 0.02% PBS-sodium azide and the samples were imaged using the Nikon A1R inverted confocal microscope.

### 2.13. Crystal Violet Staining

For crystal violet staining, ADSCs were cultured in 0.2% aNFC (1 × 10^5^ cells/ml) within TC cell culture inserts (24-well format, Ø3.0 *μ*M, 83.3932.300, Sarstedt Ltd.) for 72 h. Following cell culture and PFA fixation as detailed above, ADSCs were stained with crystal violet (0.05% *v*/*v*) for 2 h at 4°C. After incubation with the crystal violet dye, samples were washed 5 times with PBS. Images were collected using bright-field microscopy on an inverted microscope (EVOS XL, AMG, WA, USA).

### 2.14. ICC Staining

ADSCs were cultured in 0.2% aNFC (1 × 10^5^ cells/ml) within *x*-well cover glass cell culture chambers for 72 h. Following culture, media were removed and cells were gently washed with PBS, followed by fixation using 4% PFA for 20 min at RT. Subsequently, PFA was removed and cells were washed twice with PBS for 5 min. Unspecific antibody binding was blocked by incubation with 0.02% PBS-Tween with 5% goat serum for 30 min. Primary antibody against actin (1 : 250, rabbit polyclonal, Sigma-Aldrich), nestin (1 : 250 mouse monoclonal, clone #196908, R&D Systems), osteocalcin (1 : 100, mouse monoclonal, clone G-5, Santa Cruz Biotechnology Inc.), and osteopontin (1 : 100, mouse monoclonal, clone AKm2A1, Santa Cruz Biotechnology Inc.) were added in 0.02% PBS-Tween with 5% goat serum and incubated overnight at 4°C in agitation. Cells were then washed twice with PBS and incubated in PBS overnight at 4°C in agitation. PBS was replaced with the secondary antibody (1 : 300) in 0.02% PBS-Tween with 5% goat serum and incubated overnight at 4°C in agitation. Cells were then washed twice with PBS and incubated in PBS overnight at 4°C in agitation. Cells were counterstained with DAPI (1 : 2000, Sigma-Aldrich) in PBS overnight at 4°C in agitation and washed twice with PBS. Following the final wash, PBS was replaced with 0.02% PBS-sodium azide and the samples were imaged using the Nikon A1R inverted confocal microscope.

### 2.15. 3D Calcium Imaging

ADSCs were seeded in 0.2% aNFC (1 × 10^7^ cells/ml), plated within *x*-well cover glass cell culture chambers (Sarstedt Ltd.), and incubated for 72 h. The wells were rinsed with PBS and incubated with HBSS supplemented with 2.5 *μ*M Fluo-4 (Molecular Probes, Eugene, OR, USA) and 2.5 mM Probenecid for 45 min at 37°C. Subsequently, the wells were rinsed twice in PBS and incubated with HBSS+2.5 mM Probenecid for 15 min at RT before placing the chamber on an inverted fluorescence microscope (EVOS FL, AMG, WA, USA). Consecutive images of the cytosolic calcium were acquired in real time through a 4x/0.13 numerical aperture (NA) objective using a CCD camera (Sony ICX285AQ, Tokyo, Japan). The images were recorded once every 10 s for a time period of 6 min, and the average fluorescence intensity for the Ca^2+^ oscillations was analysed using ImageJ software [34].

### 2.16. Statistical Analysis

Statistical analyses were performed using GraphPad Prism software (GraphPad, La Jolla, CA, USA). Data were compared using either Student's *t*-test (two-tailed, confidence interval 95%) or one-way analysis of variance (ANOVA) with Bonferroni correction (CI 95%), where appropriate. At least 3 independent experiments were performed. *P* < 0.05 was considered statistically significant.

## 3. Results

### 3.1. aNFC Shows Low Absorption over the Whole Light Spectrum

**Figure 1:**
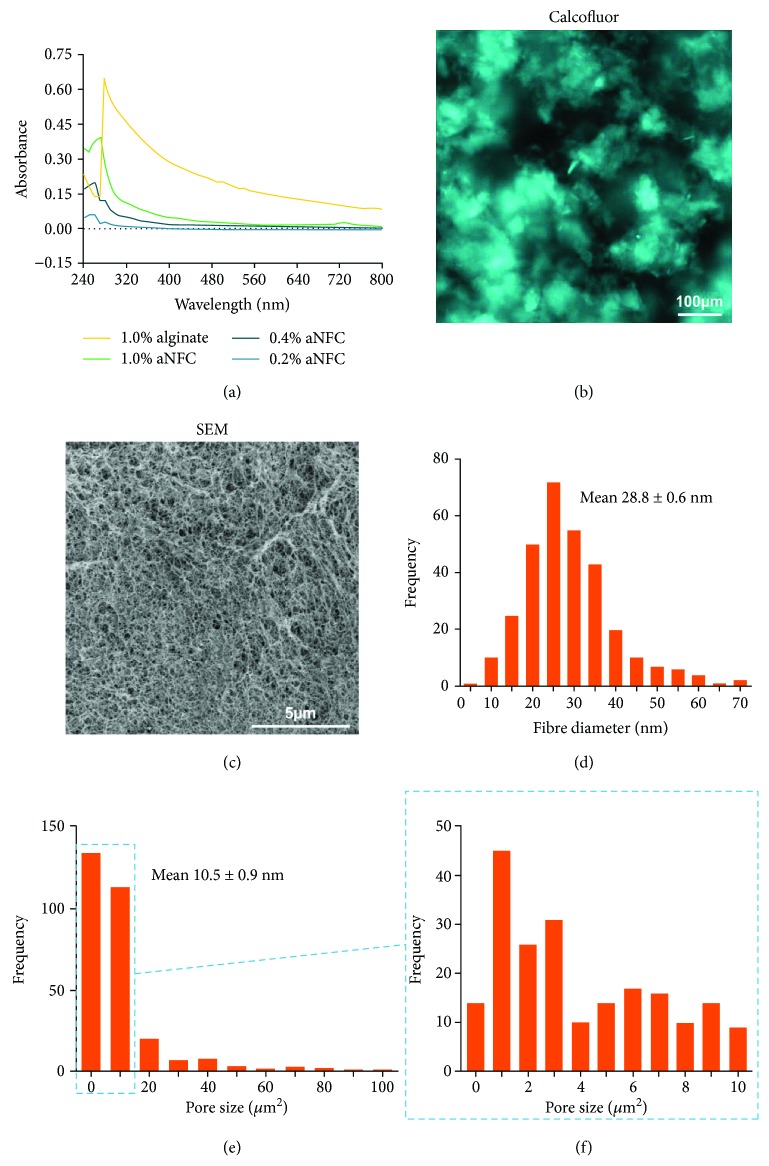
The 3D aNFC hydrogel has low light absorbance within the visible light spectrum and consists of a dense meshwork with heterogeneous pore sizes. (a) The light absorbance spectrum of 0.2%, 0.4%, and 1% aNFC and 1% alginate hydrogel revealed low absorption of aNFC between 240 and 800 nm. (b) Calcofluor white staining and fluorescence microscopy were used to visualise the distribution of the 3D aNFC hydrogel under native conditions. Highly dense and less dense areas of cellulose fibres are evenly distributed throughout the well. Bar: 100 *μ*m. (c) SEM analysis of the structural appearance and organisation of the 3D aNFC hydrogel revealed a dense, mesh-like network of nanofibres. Bar: 5 *μ*m. (d) Analysis of high-magnification SEM images revealed nanoscale fibres ranging from 10 to 70 nm with an average fibre diameter of 28.8 ± 0.6 nm. (e, f) aNFC hydrogels contain pores of heterogeneous size ranging from ~1 to 100 *μ*m with a median pore size of 10.5 ± 0.9 *μ*m^2^.

In order to assess the optical properties within the UV, visible light, and near-IR spectrum, light transmitted through 0.2%, 0.4%, and 1.0% aNFC was measured between 240 and 800 nm. As a control, 1% alginate hydrogel was used. Overall, aNFC showed low light absorbance over the whole spectrum with a slight increase at higher concentrations at wavelengths between 240 and 300 nm. Notably, even at 1%, aNFC had a lower absorption profile than the control 1% alginate hydrogel (Figure 1(a)) over the whole spectrum.

### 3.2. aNFC Forms Dense Mesh-Like Structures with Heterogeneous Pore Sizes

Calcofluor white was used to visualise the distribution of cellulose fibres within a 0.2% aNFC hydrogel. Fluorescence imaging revealed a mesh-like structure with areas of low and high cellulose content (Figure 1(b)). Subsequent analysis of the hydrogel structure using scanning electron microscopy (SEM) verified the results obtained with fluorescence microscopy. Here, a high-resolution SEM image showed a dense mesh-like network of nanofibres (Figure 1(c)), with a mean fibre diameter of 28.8 ± 0.6 nm (Figure 1(d)). Low- and high-resolution images (not shown) revealed an heterogeneous distribution of pore sizes ranging from less than 1 *μ*m^2^ to ~100 *μ*m^2^ with an average size of 10.5 ± 0.9 *μ*m^2^ (Figures 1(e)–1(f)).

### 3.3. 3D aNFC Hydrogels are Cytocompatible with MSCs

**Figure 2:**
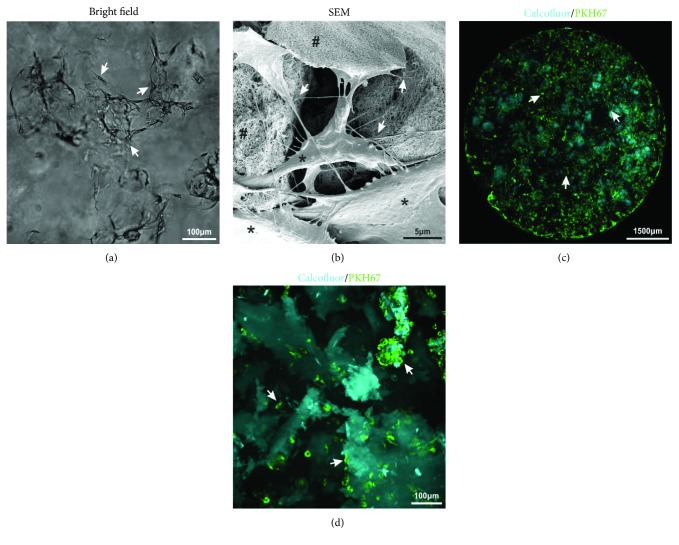
3D ADSCs interact with aNFC and are homogenously distributed. (a) ADSCs cultivated in a 0.2% aNFC hydrogel showed a MSC-typical, fibroblast-like morphology (arrows). Bar: 100 *μ*m. (b) SEM imaging of ADSCs cultured (∗) within 0.5% aNFC (#) revealed multiple cell-matrix anchor points (arrows). Bar: 5 *μ*m. (c) Spinning disc, low-magnification imaging of PKH67 fluorescently stained ADSCs (green) embedded within calcofluor-stained aNFC (cyan) showed a homogenous distribution of cells throughout the whole cell culture well (arrows). Bar: 1500 *μ*m. (d) Higher magnification spinning disc imaging demonstrating that the majority of ADSCs within the aNFC interact with the scaffold as single cells. Additionally, “nodule-like” cell aggregates were observed (arrows). Bar: 100 *μ*m.

In order to investigate the cytocompatibility with human MSCs, ADSCs were cultivated in different concentrations of aNFC (Figure 2). Bright-field microscopy (Figure 2(a)) showed adhesion and binding of MSCs to a 0.2% aNFC hydrogel in standard ADSC cultivation medium, revealing a typical, spindle-shaped, fibroblast-like morphology. SEM imaging (Figure 2(b)) demonstrated that ADSCs interact with aNFC via multiple membrane protrusions (arrows).

To examine cell distribution throughout large areas of aNFC, spinning disc imaging (Figure 2(c)) of fluorescently labelled ADSCs embedded within calcofluor-stained aNFC was performed. Subsequent image analysis revealed a homogenous distribution of the cells throughout the whole of hydrogel. Higher magnification images (Figure 2(d)) showed multiple fibroblast-like ADSCs interacting with the aNFC and several “nodule-like” cell aggregates.

### 3.4. 3D aNFC Supports High Viability of ADSCs

**Figure 3:**
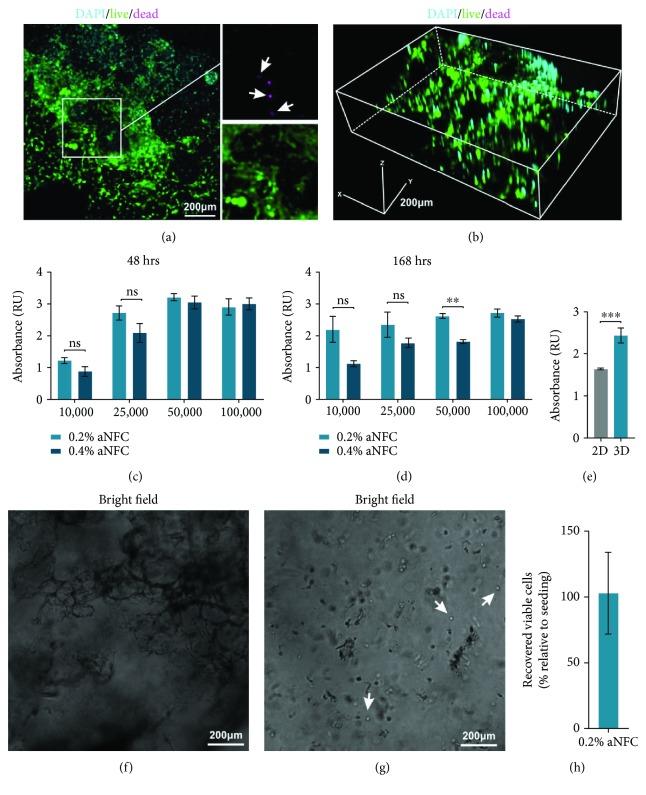
aNFC hydrogels are biocompatible with human ADSCs. (a) Confocal imaging of ADSCs within a 0.2% aNFC, stained with calcein (green, living cells) and ethidium homodimer-1 (magenta, dead cells) and counterstained with DAPI shows viable cells embedded within the aNFC, with few nonviable cells (arrows). (b) 3D reconstruction revealed even distribution of the cells in all dimensions of the aNFC. Bars in (a) and (b): 200 *μ*m. (c–e) XTT viability analyses of different concentrations of ADSCs seeded within different densities of aNFC hydrogels show high viability after 48 h (c) and 1 week (d). Direct comparison with 2D controls revealed the significantly higher viability of ADSCs cultivated in 3D (e). (f–h) Viable MSCs can be retrieved from the 3D aNFC hydrogel. ADSCs were cultivated in a 0.2% aNFC hydrogel for 48 h and retrieved by enzymatic digestion of aNFC with cellulase resulting in a single cell suspension. In total; 103 ± 31% of viable cells (h) were retrieved relative to the number of cells seeded. ^∗∗∗^*P* < 0.001 and ^∗∗^*P* < 0.01. Bars in (f) and (g): 200 *μ*m.

To assess the viability of ADSCs within the aNFC hydrogel, cells were cultured in the matrix and either assessed by Live/Dead staining or with the XTT live cell viability assay. Confocal imaging revealed that most cells within all dimensions of the hydrogel were calcein positive and thus viable (with only few cells stained with ethidium homodimer-1 (dead)) (Figures 3(a)–3(b)).

XTT-based quantitative analysis of cell viability of different concentrations of ADSCs cultivated within 0.2% and 0.4% revealed the high viability of ADSCs seeded for 48 h and 168 h at densities from 1 × 10^4^ cells through 1 × 10^5^ cells in both aNFC concentrations (Figures 3(c)–3(d)). Notably, no significant differences were seen between 0.2% and 0.4% aNFC after 48 h, whereas after 168 h, ADSCs seeded at 5 × 10^4^ cells were more viable within 0.2% aNFC compared with 0.4% (Figures 3(c)–3(d)). Next, we compared the viability of ADSCs in 0.2% with conventional 2D cell culture. Here, ADSCs cultured in 0.2% aNFC showed significantly higher viability compared to cells in 2D (Figure 3(e)).

### 3.5. Viable ADSCs Can Be Retrieved from the 3D aNFC Hydrogel

To determine whether ADSCs can be retrieved from aNFC, cells were cultivated in aNFC for 48 h followed by enzymatic hydrolysis using cellulase for 6 h followed by centrifugation. Viable cells were counted using a haemocytometer and trypan blue staining to identify nonviable cells (Figures 3(f)–3(h)). 103 ± 31% of viable cells were retrieved relative to the number of cells seeded.

### 3.6. 3D aNFC Hydrogels Are Suitable for Osteogenic and Adipogenic Differentiation of ADSCs in 3D

**Figure 4:**
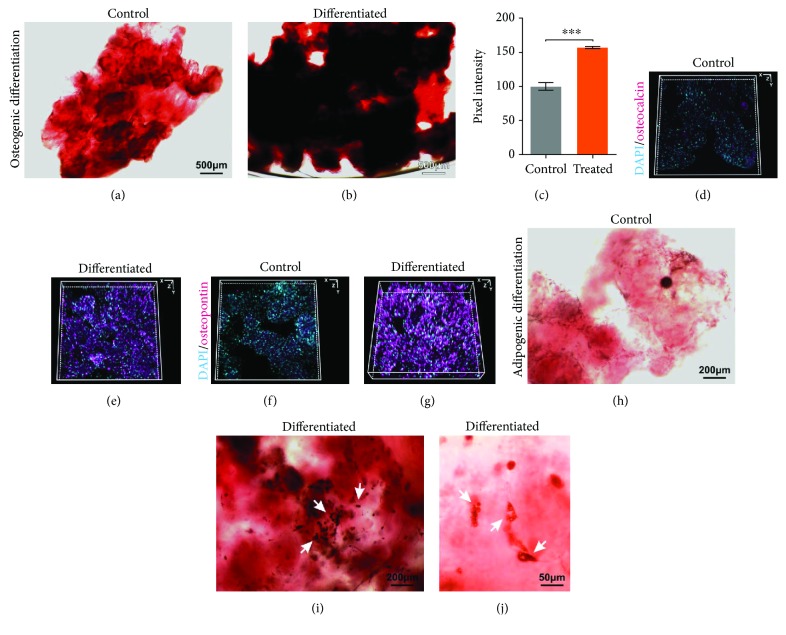
ADSCs can undergo osteogenic and adipogenic differentiation in 3D. (a, b) ADSCs were subjected to control or osteogenic differentiation treatments in 0.2% aNFC for 21 days and subsequently stained for calcium deposition with Alizarin Red S. Images of differentiated ADSCs (b) clearly show calcium deposition (red), whereas no differentiation was observed in control cells (a). Bars in (a) and (b): 500 *μ*m. (c) Image analysis of calcium deposition showed significantly higher calcium deposition within differentiated cells compared to the control. (d–g) Immunocytochemical stainings with antiosteocalcin (d, e) and antiosteopontin antibodies (f, g) reveal strong immunopositivity in the osteogenically differentiated cells (e, g) compared to control cells (d–f). (h–j) ADSCs were subjected to adipogenic differentiation within 0.2% aNFC for 21 days. Following Oil Red O staining, lipid deposition was observed in treated cells (arrows in (i) and (j)) compared with the untreated control (h). Bars in (h) and (i): 200 *μ*m; bar in (j): 50 *μ*m.

To assess the differentiation capacity of ADSCs within 3D aNFC, cells were subjected to differentiation for 21 days. Following osteogenic differentiation, calcium deposition was assessed by Alizarin Red S staining (Figures 4(a)–4(c)), whilst expression of the osteogenic markers osteocalcin and osteopontin was investigated by immunocytochemistry and subsequent 3D reconstruction of images acquired by confocal laser scanning microscopy (Figures 4(d)–4(g)). Here, we were able to demonstrate that ADSCs within aNFC deposit significantly higher amounts of calcium after 21 days of differentiation compared to undifferentiated 3D controls (Figure 4(c)). In accordance with the results of the Alizarin Red S staining, immunocytochemistry for osteocalcin and osteopontin revealed high expression of both markers in differentiated cells only. Adipogenesis was assessed by Oil Red O staining (Figures 4(h)–4(j)). Lipid-rich vacuoles were observed within the adipogenic differentiated cells, whereas no lipid droplets were found in undifferentiated cells (Figures 4(h)–4(j)).

### 3.7. aNFC Allows for Easy Light Microscopy of ADSCs in 3D

**Figure 5:**
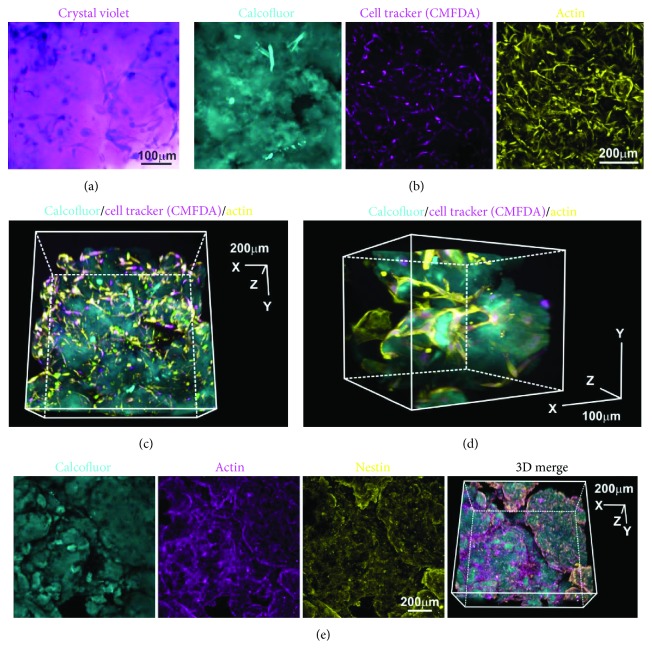
aNFC is compatible with multiple light microscopy-based assays. (a) ADSCs were cultivated within aNFC and stained using crystal violet. Bright-field microscopy revealed evenly distributed, dark-stained ADSCs. Bar: 100 *μ*m. (b) ADSCs embedded in 0.2% aNFC were stained using cell tracker CMFDA (magenta) and phalloidin (yellow). aNFC was counterstained with calcofluor. A maximum intensity projection is shown. Bar: 200 *μ*m. (c, d) 3D reconstruction demonstrated even staining intensity in all dimensions of the aNFC hydrogel. Bar in (c): 200 *μ*m; bar in (d): 100 *μ*m. (e) Immunocytochemistry with subsequent confocal microscopy was applied to visualise actin and the intermediate filament nestin in ADSCs in 0.2% aNFC. Bar: 200 *μ*m.

In order to visualise ADSCs in 3D using a nonfluorescent dye, cells cultivated in aNFC were subjected to crystal violet staining. Microscopy demonstrated that ADSCs in aNFC can be visualised by crystal violet as evidenced by the intense violet staining of the cells revealing a typical fibroblastic morphology (Figure 5(a)). In order to assess the compatibility of aNFC with ADSCs stained with fluorescent dyes emitting at different wavelengths, cells were cultivated with a 0.2% aNFC hydrogel and fixed and stained with cell tracker CMFDA and phalloidin, whereas aNFC was counterstained with calcofluor. Subsequent confocal laser scanning microscopy revealed homogenously labelled ADSCs in all dimensions of the hydrogel with low background fluorescence in all channels (Figures 5(b)–5(d)). Notably, the morphological appearance of ADSCs revealed by the 3D reconstruction of the confocal images was consistent with that seen by SEM. Next, we studied the compatibility of aNFC with indirect immunocytochemical (ICC) staining protocols. ADSCs within aNFC were fixed and stained with primary antibodies against actin and nestin followed by incubation with secondary, fluorochrome-conjugated antibody and aNFC staining using calcofluor. Confocal microscopy and 3D reconstruction demonstrated that ICC staining of cells is feasible within 3D aNFC with good signal-to-noise ratio in all channels (Figure 5(e)).

### 3.8. Spontaneous Cytosolic Ca^2+^ Oscillations in ADSCs Can Be Imaged and Quantified within 3D aNFC

**Figure 6:**
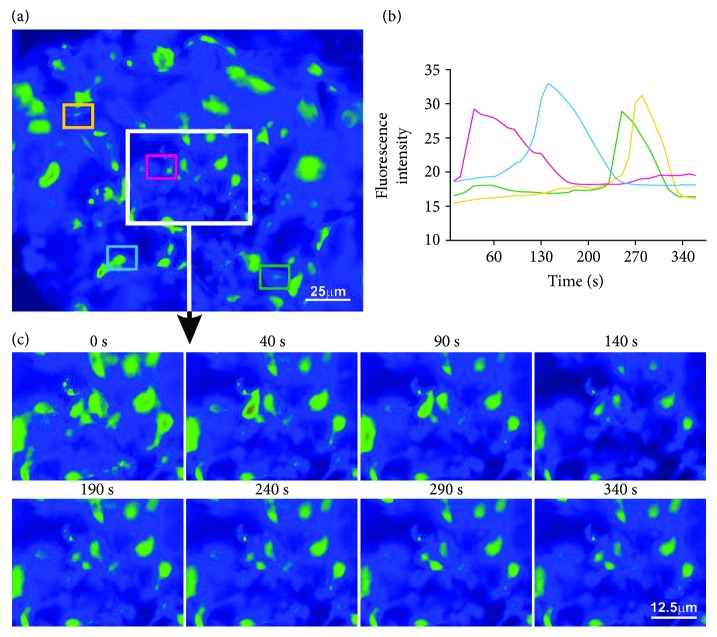
aNFC allows calcium imaging in 3D. (a) Fluorescence time lapse images were collected for 6 min, recording spontaneous cytosolic calcium oscillations in ADSCs within 0.2% aNFC. The fluorescence intensity of four individual ADSCs was quantified (coloured boxes). (b, c) Quantification of fluorescence intensity (b) shows unsynchronized cytosolic calcium oscillations (c). Bar in (a): 25 *μ*m; bar in (c): 12.5 *μ*m.

Calcium oscillations are an intrinsic property of undifferentiated MSCs [35, 36], and their electrical manipulation is known to have a positive impact on osteogenic differentiation. Thus, calcium imaging is an important tool in studying MSC biology. In order to assess the feasibility of calcium imaging of ADSCs in 3D, cells were cultivated in 0.2% aNFC and incubated with Fluo-4 dye. To record the spontaneous cytosolic calcium oscillations, fluorescence time lapse imaging was performed (Figure 6). Data analysis revealed unsynchronized cytosolic calcium oscillations (Figures 6(b) and 6(c)) consistent with the data obtained in 2D (data not shown).

## 4. Discussion

This study details for the first time the properties and cell culture application of a newly developed anionic form of a plant-derived nanofibrillar cellulose hydrogel. We showed that aNFC has low light absorbance over the whole visible light spectrum and that its 3D structure features nanoscale fibres and pores of different sizes compatible with cell infiltration and proliferation. Moreover, similar to conventional NFC hydrogels [31], aNFC is biocompatible with human ADSCs. Cells within aNFC show extensive cell-matrix anchor points, have a typical fibroblast-like morphology, and are homogenously distributed in all three dimensions of the hydrogel. ADSCs remained viable within the aNFC up to 3 weeks of culture and could be retrieved following enzymatic digestion of the hydrogel. Additionally, ADSCs were successfully differentiated along the osteogenic and adipogenic lineages in 3D.

MSCs including ADSCs represent one of the most promising and widely used cell types in regenerative medicine with the current view that paracrine action is the most likely therapeutic mode of action by which they contribute to tissue regeneration. However, since the generation of the conditioned medium and isolation of extracellular vesicles relies on high cell numbers to achieve clinically relevant outcomes, expansion of cells requires prolonged cell culture and represents a significant bottleneck towards clinical application. In this context, it has been demonstrated that extended 2D cultivation of various adult stem cells can lead to chromosomal aberrations as well as to tumorigenic transformation of the cells [11, 12, 37]. This can be at least partly attributed to the removal of the stem cells from their endogenous niche. Changes to cell shape and overall geometry [38], cytoskeletal flattening, and the associated changes to the nuclear shape [39] during 2D cell culture have also been shown to affect the gene [40] and protein expression profile [41], as well as their proliferation pattern [42] and differentiation capacity [43].

Notably, Yang et al. [44] were able to demonstrate that 3D cultivation of MSCs increases autophagy and associated repair mechanisms. Therefore, cultivation and expansion and retrieval of MSCs in 3D could reduce the risk of tumorigenic transformation during expansion *in vitro*, providing a more stable microenvironment for isolated MSCs *ex vivo*, reducing cellular senescence, maintaining differentiation capacity, and increasing the yield of cells with a high proliferation capacity.

The high costs of clinical grade reagents and cell culture media represent an often neglected aspect in the translation of basic stem cell biology into the clinics. Here, 3D cell culture has the obvious advantage of a significantly higher surface area in comparison to 2D [45] allowing high-density cell culture with lower volumes of medium, thus reducing the costs of cell expansion [46].

Currently, there is a multitude of natural 3D hydrogels suitable for mammalian cell culture systems including but not limited to alginate, fibrin, collagen, and Matrigel™ [15, 16, 19, 20, 22]. However, most of the commercially available, natural hydrogels are animal derived, require cross-linking, or do not allow retrieval of the cells after cell culture without the use of enzymes that could affect mammalian cells. To address these issues, we and others investigated the biocompatibility of plant-derived, standardised nanofibrillar cellulose (NFC) hydrogels with different cell types including induced pluripotent stem cells, hepatic cells, neural crest-derived stem cells, and MSCs [31, 47–49]. In our previous study, we demonstrated that NFC is biocompatible with human MSCs and neural crest-derived stem cells, without having a negative impact on their osteogenic differentiation capacity [31].

Moreover, we showed that NFC allows simple cell retrieval using cellulase.

Notably, despite all the advantages of 3D over 2D cell culture, the imaging of cells embedded within 3D hydrogels is challenging and is often hampered by the opacity of the scaffolds. This is a consequence of spatial gel inhomogeneity in many hydrogels including alginate hydrogels (Figure 1(a)) [30].

In the present study, we demonstrate that aNFC has low light absorption in the visible light spectrum and allows for conventional and fluorescence microscopy with good signal-to-noise ratios. We further demonstrate that human ADSCs interact with the aNFC being evenly distributed within all three dimensions of the hydrogel and that they are viable with both 0.2% and 0.4% aNFC solid weight content for culture periods up to 168 h (Figure 3(d)). Although no signs of apoptosis were observed in ADSCs differentiated in aNFC for 21 days, future studies including an in-depth analysis of cell viability and proliferation over prolonged time periods need to be carried out to assess the cell behaviour during long-term exposure of cells to aNFC.

We used Alizarin Red S and immunocytochemistry to demonstrate that aNFC supports osteogenic differentiation in 3D. In addition, Oil Red O staining revealed that aNFC does not interfere with the adipogenic cell fate. However, future studies need to assess this cell fate at the mRNA and protein level using specific markers of adipogenesis, e.g., LPL and PPAR-*γ*.

Finally, we show that aNFC is compatible with fluorescence live cell microscopy as demonstrated by the visualisation of calcium oscillations in MSCs in 3D (Figure 6).

## 5. Conclusion

Overall, our study indicates that the optically transparent aNFC supports culture expansion and differentiation of human ADSCs whilst allowing for an enzymatic retrieval of viable cells. Thus, aNFC combines the advantages of other plant-derived NFC hydrogels including good biocompatibility, freedom of animal components, batch-to-batch consistency, and easy retrieval of the cells with excellent optical properties providing a platform for the expansion and analysis of cells in 3D.

## Data Availability

The data used to support the findings of this study are included within the article.
